# Evolutionary Strategies in Nanocomposite Proton Exchange Membranes: A Physical Chemistry Applied Materials (PCAM) LAB Review on Material Design, High-Temperature Performance, and Durability

**DOI:** 10.3390/polym17233185

**Published:** 2025-11-29

**Authors:** Isabella Nicotera, Luigi Coppola, Cataldo Simari

**Affiliations:** Department of Chemistry and Chemical Technologies, University of Calabria, 87036 Rende, Italy

**Keywords:** PEMFCs, proton exchange membranes, nanocomposite membranes, nanomaterials, PCAM lab, advanced characterization

## Abstract

Polymer Electrolyte Membrane and Direct Methanol Fuel Cells (PEMFCs/DMFCs) are vital clean energy technologies, yet their adoption is hindered by limitations in industry-standard PFSA membranes. PFSA degrades above 80 °C, suffers substantial methanol crossover, and contains environmentally persistent PFAS, which raises significant environmental and cost concerns due to its persistence and bioaccumulation, driving a global imperative for sustainable, fluorine-free alternatives. In response to these challenges, the PCAM Lab has dedicated extensive research efforts to developing advanced PEMs. A primary focus is non-fluorinated alternatives (NFPs), including sulfonated Polysulfone (sPSU) and Sulfonated polyether ether ketone (sPEEK), which have emerged as a compelling, cost-effective, and environmentally friendly alternative to the PFSA benchmark. Beyond NFPs’ intrinsic advantages, the lab’s implementation of nanocomposite strategies, involving the incorporation of various functional nanofillers, has proven transformative. This report provides a comprehensive, critical analysis of the state of the art in PEM research, contextualizing the specific contributions of the Physical Chemistry Applied Materials (PCAM) Lab within the broader global scientific dialog. While the PCAM Lab has made notable strides in utilizing Sulfonated Polysulfone (sPSU) and nanocomposite strategies, a true assessment of the field requires integrating these findings with the seminal works of leading international research groups. By synthesizing data on sulfonated polyphenylenes, advanced graphene architectures, and industrial manufacturing constraints, this analysis illuminates the divergent pathways currently being explored to overcome the “Nafion Dilemma”.

## 1. Introduction to Polymer Electrolyte Membranes in Fuel Cells

### 1.1. Fundamental Role of PEMs in Fuel Cell Operation

The contemporary geopolitical and environmental landscape is defined by a singular, urgent imperative: the decarbonization of the global energy infrastructure. As nations grapple with the escalating consequences of anthropogenic climate change, the transition from combustion-based power generation to electrochemical energy conversion has graduated from a scientific curiosity to a critical industrial necessity. Within this macro-strategic framework, Hydrogen Fuel Cells, specifically Polymer Electrolyte Membrane Fuel Cells (PEMFCs), and Direct Methanol Fuel Cells (DMFCs) have emerged as pivotal technologies. These devices provide a pathway to sustainable mobility and stationary power that is decoupled from the carbon cycle, offering high efficiency and zero tailpipe emissions [1,2,3]. As the hydrogen economy matures, the demands on the central component of these devices, the Proton Exchange Membrane (PEM), have escalated from simple ion conduction to a complex set of multi-objective requirements: operation at elevated temperatures (>100 °C), tolerance to low humidity, mechanical robustness under dynamic load cycling, and, increasingly, freedom from per- and polyfluoroalkyl substances (PFAS) [4]. Their primary function is to facilitate the selective transport of protons from the anode, where fuel is oxidized, to the cathode, where oxygen is reduced [5]. Simultaneously, PEMs must act as an impermeable barrier to reactant gases (such as hydrogen, oxygen, and methanol) and electrons. This dual role is essential to prevent short-circuiting and ensure efficient electrochemical energy conversion, which directly impacts the overall efficiency, power density, and longevity of the fuel cell system. The optimal PEM must achieve the dual function of facilitating efficient proton transport from anode to cathode, while simultaneously acting as an impermeable barrier to reactant gases (H_2_, O_2_) and liquid fuel (methanol). The overall efficiency, power density, and operational lifespan of the fuel cell system are directly contingent upon the PEM’s performance in this dual role [6].

While the industry standard, Nafion, offers high proton conductivity and chemical stability under ideal conditions, its operational limitations, i.e., performance collapse above 80 °C and excessive methanol crossover, impose significant system-level penalties [7,8,9]. These drawbacks necessitate intricate thermal and water management systems, which in turn increase the overall Balance-of-Plant (BoP) costs, thereby fundamentally hindering the large-scale commercialization of PEM technologys.

In Direct Methanol Fuel Cells (DMFCs), the permeation of methanol from the anode to the cathode through the membrane, known as methanol crossover, constitutes a severe drawback [10]. This phenomenon results in mixed potential at the cathode, significantly reducing fuel efficiency and overall cell voltage. It also contributes to cathode catalyst poisoning and compromises the long-term stability of the fuel cell. Nafion’s inherent high permeability to methanol exacerbates this issue, making it a primary challenge for DMFC development.

Furthermore, the environmental persistence and bioaccumulation of the perfluorinated compounds (PFAS) inherent to Nafion’s structure constitute a critical environmental and regulatory constraint, driving the global imperative for sustainable, fluorine-free alternatives [11]. These chemicals are environmentally persistent and bioaccumulative, leading to a strong push within the scientific community to move away from fluorinated polymers. The disposal of Nafion is a potential source of toxic perfluorocarboxylic acids, which are widespread globally and found in human blood, associated with adverse health effects [11]. The materials science community is thus faced with a multi-objective optimization challenge: new PEM materials must not merely match or slightly exceed Nafion’s performance but must simultaneously offer significant advantages in four critical areas: performance (especially at high temperature/low humidity), cost-effectiveness, environmental benignity, and, crucially, long-term durability.

### 1.2. Rationale for Alternative Materials and Nanocomposite Strategies

In light of the substantial material-level limitations of Nafion, particularly dehydration and methanol crossover, which translate directly to system-level consequences (increased complexity and cost), the focus of research has shifted toward developing robust, cost-effective, non-fluorinated alternatives. Sulfonated aromatic polymers, such as sulfonated polysulfone (sPSU) and sulfonated polyether ether ketone (sPEEK), have emerged as promising candidates due to their chemical tunability, excellent thermal stability, and low environmental impact [4]. To overcome the inherent weaknesses of these hydrocarbon host polymers (specifically, excessive swelling and compromised mechanical stability at high degrees of sulfonation), the PCAM Lab, among others, has focused extensively on nanocomposite strategies. This approach involves the strategic incorporation of inorganic or organic nanofillers to achieve a synergistic amalgamation of properties. The rationale is two-fold: first, to introduce hygroscopic sites or conductive pathways that maintain performance under dehydrating conditions, and second, to mechanically reinforce the polymer matrix, thereby restricting chain movement and improving dimensional and thermal stability. Successful PEM development requires materials capable of inherently managing water, tolerating impurities, and operating under broader temperature and humidity ranges, thus enabling a simpler, more robust, and more cost-effective overall fuel cell system [12].

The problem statement consistently links Nafion’s material-level limitations, such as dehydration and methanol crossover, directly to system-level consequences like increased complexity and cost. This highlights that the development of successful PEMs is not merely a material science challenge but a systemic engineering hurdle. Achieving optimal PEM performance is not just about improving isolated material properties but about enabling a simpler, more robust, and more cost-effective overall fuel cell system. This holistic perspective drives the search for materials that can inherently manage water, tolerate impurities, and operate under broader conditions, thereby reducing the need for complex external balance-of-plant components [13].

Furthermore, Nafion is acknowledged as the “state-of-the-art” in terms of performance but is simultaneously criticized for its high cost and environmental impact [11]. This presents a fundamental dilemma for commercialization and widespread adoption. Future PEM innovation must navigate a complex multi-objective optimization landscape. It is no longer sufficient for new materials to merely match or slightly exceed Nafion’s performance; they must also offer significant advantages in terms of cost-effectiveness, environmental benignity (e.g., non-fluorinated), and scalability to meet the demands of a sustainable energy future.

### 1.3. Overview of the PCAM Lab’s Research Trajectory

This review synthesizes the PCAM Lab’s trajectory over the past decade in developing advanced nanocomposite proton exchange membranes. The research methodology is characterized by a multi-scale, synergistic approach, explicitly integrating advanced computational modeling (Molecular Dynamics, Density Functional Theory) with extensive experimental characterization (PFG-NMR, EIS, DMA). This approach provides a fundamental understanding of the structure-performance relationships, enabling the rational design of materials at the molecular scale and nanoscale to achieve desired macroscopic properties. The review highlights the lab’s innovations in materials design, multi-scale characterization, and manufacturing strategies, specifically focusing on sPSU and sPEEK-based systems enhanced with Layered Double Hydroxides (LDH), graphene derivatives, and organosilica layered materials.

## 2. Foundational Ionomer Chemistry: Structure, Synthesis, and Inherent Properties

To properly address the performance characteristics and intrinsic limitations of the membranes developed, it is necessary to establish the chemical foundation of the main ionomers studied by the PCAM Lab.

### 2.1. Perfluorosulfonic Acid (PFSA) Ionomers: The Nafion Benchmark

**Figure 1:**
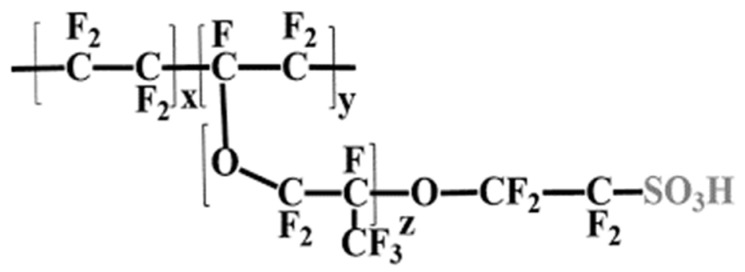
Chemical structure of Nafion.

PFSA ionomers, such as the quintessential Nafion^®^, are characterized by a polytetrafluoroethylene (PTFE) backbone—a robust, inert perfluorinated structure (CF_2_CF_2_)—to which terminal sulfonic acid functional groups (-SO_3_H) are attached via long perfluorinated side chains (see Figure 1). The extreme chemical stability and inertness of the C-F bonds in the backbone are what grant Nafion its superior chemical durability, enabling it to resist attack by Reactive Oxygen Species (ROS) generated during fuel cell operation. The high acidity of the sulfonic groups, coupled with the nanoscopic phase separation between the hydrophobic PTFE backbone and the hydrophilic ionic clusters, facilitates high proton conductivity. However, the architecture dictates its operational vulnerability. The performance relies critically on the precise structure of the hydrophilic channels, which are maintained by water molecules. Above 80 °C, the evaporation of water disrupts these channels, leading to a performance collapse.

### 2.2. Aromatic Hydrocarbon Ionomers: Sulfonated Polysulfone (sPSU) and Sulfonated Polyether Ether Ketone (sPEEK)

**Figure 2:**
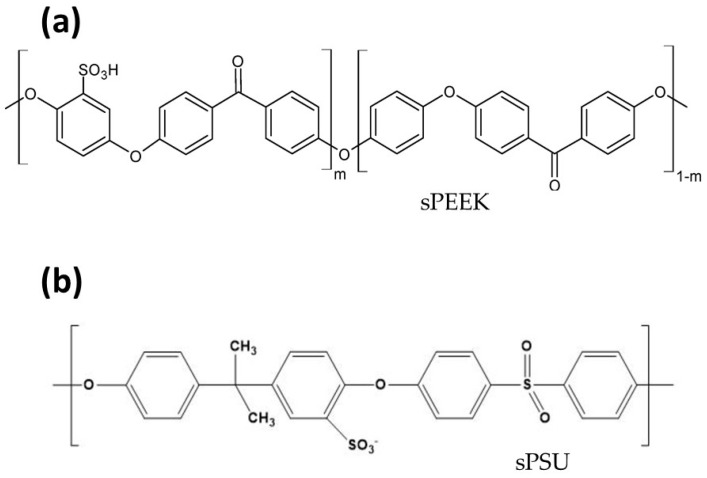
Chemical structure of (**a**) sulfonated poly (ether-ether-ketone) (sPEEK) and (**b**) sulfonated polysulfone (sPSU).

The primary alternative PEMs explored by the PCAM Lab are based on aromatic hydrocarbon polymers, specifically Sulfonated Polyether Ether Ketone (sPEEK) Sulfonated Polysulfone (sPSU), whose chemical structures are schematized in Figure 2. sPSU is synthesized by sulfonating the commercially available polysulfone backbone [14]. The resulting ionomer is a compelling, cost-effective, and environmentally friendly alternative, known for its excellent film-forming ability and notable thermo-mechanical resistance. With regard to sPEEK, it has to be considered that PEEK itself is electrically non-conductive but becomes an effective ionomer after sulfonation, which introduces sulfonic acid groups (-SO_3_H) onto the aromatic rings [15]. While possessing excellent thermal and chemical stability, the proton conductivity of sPEEK is highly tunable, increasing directly with the degree of sulfonation (DS). In this regard, a fundamental trade-off exists in all aromatic hydrocarbon ionomers: increasing the DS to maximize proton conductivity simultaneously leads to excessive water uptake, catastrophic swelling (sPEEK swelling ~200% after 10 days into water), and the subsequent deterioration of mechanical and chemical stability [16]. This instability, primarily manifested by channel collapse and physical weakening, restricts the commercial application of high-DS pristine hydrocarbon membranes. Furthermore, a critical difference between Nafion and aromatic polymers is the location of their functional groups. The aromatic C-H bonds in the hydrocarbon backbone are inherently susceptible to chemical degradation via nucleophilic attack by Reactive Oxygen Species (ROS, e.g., hydroxyl radicals, i.e., •OH) generated during fuel cell operation. This makes the vulnerability of the aromatic backbone to chemical degradation the primary durability challenge. Consequently, the incorporation of nanofillers in hydrocarbon systems is not merely a performance enhancement strategy but an absolute necessity for physical shielding and chemical stabilization to ensure long-term operational viability.

## 3. PCAM Lab’s Nanocomposite Strategies for Enhanced PEM Performance

The development of nanocomposite membranes has represented a pivotal strategy in the PCAM Lab to overcome the inherent limitations of pristine polymer electrolytes. By incorporating various functional nanofillers, researchers aim to synergistically enhance critical properties such as proton conductivity, water management, mechanical integrity, and barrier properties against fuel crossover.

### 3.1. Layered Double Hydroxides (LDH) as Multi-Functional Nanoclinckers

The incorporation of LDH into polymer matrices leads to a cascade of enhancements driven by specific mechanisms.

**Figure 3:**
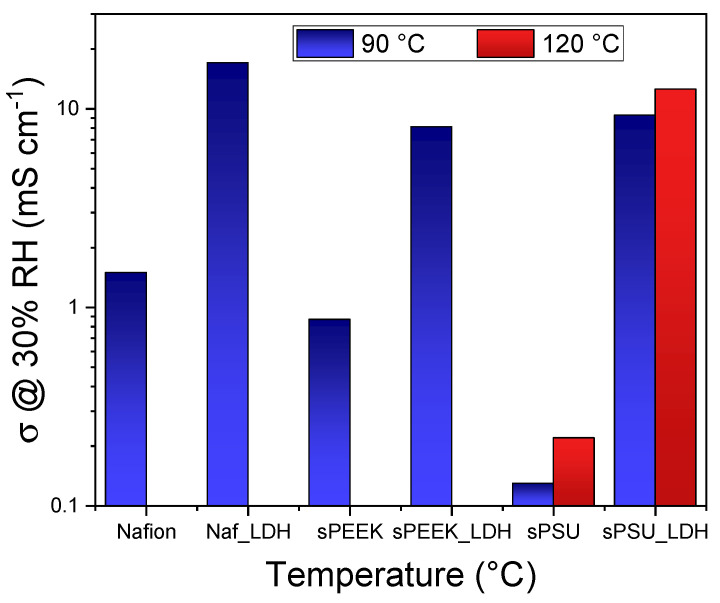
Performance comparison of proton conductivity for bare polymers and corresponding LDH-based nanocomposite. For all the systems, the values at optimal filler loading are reported. IEC Nafion: 0.94 meq/g, IEC sPEEK: 1.91 meq/g, IEC sPSU: 1.39 meq/g.

- **Improved Water Molecular Dynamics and Retention**: LDH incorporation significantly enhances water retention, particularly at elevated temperatures. For instance, sPSU/LDH membranes can retain up to 40 wt% water content even at 130 °C, a stark contrast to pristine sPSU, which becomes almost dehydrated under similar conditions [17]. This impressive water retention is attributed to strong electrostatic interactions between the LDH platelets and water molecules, especially the “bound water” population, which resists evaporation even at high temperatures. These enhanced interactions lead to significantly higher water self-diffusion coefficients (D) in LDH composites. For example, at 130 °C, the water self-diffusion coefficient for sPSU/LDH is 6.63 × 10^−6^ cm^2^s^−1^, which is more than an order of magnitude higher than that of pristine sPSU (1.31 × 10^−7^ cm^2^s^−1^). These improved water dynamics are crucial for maintaining efficient proton transport in dehydrating environments [18].
- **Enhanced Dimensional Stability**: LDH platelets act as physical crosslinkers between adjacent polymer chains. This strong electrostatic interaction restricts the polymer chains from expanding excessively, thereby significantly improving dimensional stability. For sPSU/LDH, volume swelling is almost negligible with increasing temperature compared to pristine sPSU, which exhibits massive swelling [17]. This enhanced stability is further corroborated by an increase in the glass transition temperature (Tg) of the hydrophilic clusters, for example, from 200 °C for sPSU to 225 °C for sPSU/LDH. This physical crosslinking effectively re-engineers the polymer’s ionic network, preventing channel collapse at high temperatures and restricting swelling.
- **Increased Proton Conductivity (Grotthuss Mechanism)**: LDH nanoplatelets directly contribute to proton transport, boosting both the vehicular and Grotthuss mechanisms, independently from the hosting matrix (see Figure 3). They connect isolated sulfonic acid groups, effectively filling gaps and generating continuous networks for proton migration, which is crucial under dehydrating conditions where the Grotthuss mechanism dominates. The activation energy for proton conductivity decreases significantly upon LDH incorporation, for instance, from 16.10 kJ mol^−1^ for sPSU to 9.25 kJ mol^−1^ for sPSU/LDH, indicating a noticeable improvement in the efficiency of proton conduction [17].
- **Methanol Crossover Mitigation**: LDH composites significantly reduce methanol permeability by increasing the tortuosity of diffusional paths within the membrane. For sPSU/LDH, the methanol crossover current can be as much as 60% lower than Nafion 212 and 25% lower than bare sPSU [19]. This is primarily due to the physical cross-linking action of LDH, which reduces the effective size of hydrophilic channels, making it more difficult for larger methanol molecules to permeate. This selective hindering of larger methanol molecules, while facilitating smaller protons, is a key advantage.
- **Intermediate-Temperature PEMFC Performance**: sPSU/LDH membranes exhibit superior proton conductivity, especially at low humidity. For example, sPSU/LDH can achieve 4 mS cm^−1^ at 90 °C and 20% RH, which is 20-fold higher than pristine sPSU and, critically, explicitly exceeds the performance benchmark of Nafion 212 (2.8 mS cm^−1^) under the same challenging dehydrating conditions. This quantitative advantage demonstrates the material’s ability to successfully overcome the intrinsic dehydration collapse typical of perfluorinated membranes above 80 °C. In single H_2_/O_2_ fuel cell tests, sPSU/LDH3 demonstrates a peak power density of 254 mW cm^−2^ at 80 °C/30% RH, representing a 30% improvement over Nafion recast/212 [17]. Furthermore, it maintains a robust 204.5 mW cm^−2^ at 110 °C/25% RH, with only a 20% reduction in power compared to 40–50% reductions observed for Nafion and pristine sPSU. This highlights its effective self-humidification capability and significant potential for high-temperature operation.
- **DMFC Performance**: sPSU/LDH achieves a remarkable power density of 150 mW cm^−2^ at 80 °C in 5 M methanol solution, making it the highest among the tested membranes [19]. This performance underscores its superior chemical and dimensional stability, enabling extended DMFC operation under high methanol concentrations where Nafion struggles due to excessive swelling.
- **Durability and Stability Metrics:** The viability for practical application is strongly supported by the inherent durability proxies exhibited by the sPSU/LDH composites. The incorporation of LDH platelets acts as a highly effective physical crosslinker, yielding substantial mechanical and thermal stability improvements. This is evidenced by a significant increase in the glass transition temperature (Tg) of the hydrophilic clusters, shifting from 200 °C for pristine sPSU to 225 °C for sPSU/LDH. Furthermore, Dynamic Mechanical Analysis (DMA) confirms that the storage modulus (E’) is almost 80% higher than the bare polymer [17]. This pronounced mechanical reinforcement ensures negligible volume swelling under increasing temperature. The combined stability gains, i.e., reduced swelling and increased Tg, provide robust evidence of the membrane’s resistance to the dominant mechanical degradation modes (cracking, thinning) induced by the hydration/dehydration cycles typical of high-temperature fuel cell operation, significantly strengthening the case for long-term commercialization potential. This demonstrates the material’s structural resistance to fatigue, addressing the durability imperative required for practical application.
- **Anisotropy via Alignment**: Mechanical alignment of LDH nanoparticles, for instance, through doctor blade casting, can induce anisotropy in the membrane’s properties. While this can lead to higher in-plane conductivity, through-plane conductivity might be hindered due to a “blocking effect” from the aligned lamellae [20]. This understanding has led to the development of sophisticated dual-layer hybrid membranes. These designs strategically combine an aligned layer for enhanced methanol barrier properties with a cast layer for efficient proton transport, achieving impressive power densities (e.g., 300 mW cm^−2^ at 100 °C). This represents a sophisticated architectural design principle for PEMs, where researchers deliberately manipulate filler orientation to achieve specific performance profiles, moving beyond simple material addition to deliberate membrane engineering at the macroscopic level.

### 3.2. Graphene-Based Nanofillers: Engineering Proton Superhighways

**Figure 4:**
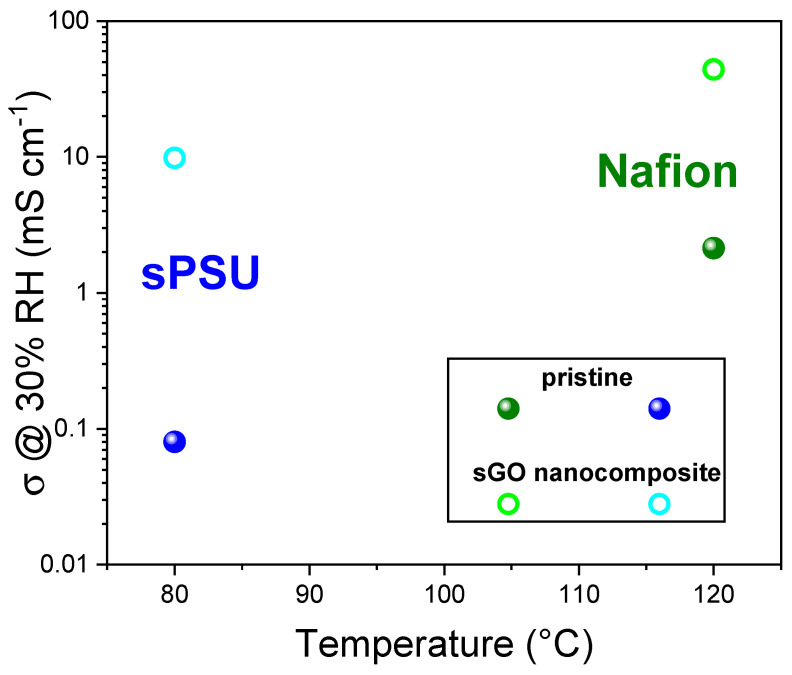
Proton conductivity of Nafion and Nafion-sGO (at 120 °C) and for sPSU and sPSU-sGO. For all the systems, the values at optimal filler loading are reported. IEC Nafion: 0.94 meq/g, IEC sPSU: 1.39 meq/g.

Graphene-based nanofillers, extensively studied by the PCAM Lab, have emerged as highly effective additives for enhancing the performance of both sPSU and Nafion membranes. These materials act as internal humidifiers and proton superhighways, structuring water into a thermally stable “bound” state that resists evaporation and remains mobile. The extended 2D (graphene) or 1D (CNT) networks, when functionalized, provide continuous, low-resistance pathways for proton hopping (Grotthuss mechanism), effectively bypassing the limitations of the polymer’s inherent ionic channels. This represents a fundamental shift from merely adding hydrophilic sites to engineering a resilient, interconnected proton-conducting network within the membrane. **Sulfonated graphene oxide (sGO),** an organo-sulfonated derivative of graphene oxide synthesized via chemical grafting [21], significantly increases hydrophilicity and interlamellar distance. sGO exhibits outstanding water retention, maintaining high water self-diffusion coefficients up to 130 °C (e.g., 1.5 × 10^−5^ cm^2^s^−1^ at 130 °C for Nafion-sGOsulf) in conditions where unmodified GO or pristine Nafion typically fail due to dehydration [22]. The electrochemical performance of Nafion-sGOsulf composites shows significantly enhanced proton conductivity. For instance, Nafion-sGOsulf can achieve 231.9 mS cm^−1^ at 120 °C and 90% RH, representing an 81% enhancement over pristine Nafion. Even under low relative humidity (30%), Nafion-sGOsulf maintains high conductivity (44.9 mS cm^−1^ at 90 °C), a direct consequence of its superior water retention and the direct involvement of sGO nanoplatelets in proton hopping along hydrogen bonding networks. Furthermore, sGO has demonstrated the ability to reduce methanol crossover in DMFCs and increase cell power density [21]. Similar outstanding results were registered in sPSU-based nanocomposites [23] with a conductivity improvement of almost two orders of magnitude in the low-humidity regime, as shown in Figure 4.

### 3.3. Organosilica Layered Materials (sSLM, PSLM): Designer Nanofillers

Organosilica layered materials, a focus of the PCAM Lab’s research, represent a class of designer nanofillers where the surface chemistry is specifically tuned to create optimal interfacial compatibility and electrostatic interactions with the chosen polymer matrix. This leads to a polymer-filler-specific synergy that dictates the ultimate performance, emphasizing that a “one-size-fits-all” filler approach is inefficient and that rational design based on polymer chemistry is key.

- **Sulfonated Siliceous Layered Materials (sSLM)** are synthesized via a one-pot sol–gel process using 3-(trihydroxysilyl)propyl-1-propane-sulfonic acid, yielding a layered material with a high density of sulfonic groups [24]. Incorporation into PFSA leads to synergistic enhancements of electrochemical performance and thermomechanical stability. Both the Ion Exchange Capacity (IEC) and water uptake increase (e.g., IEC from 0.94 to 1.23 meq/g; water uptake from 24 to 32 wt% at 5% filler loading). N-sSLM5 (Nafion with 5% sSLM) consistently shows the highest water self-diffusion coefficients across the entire temperature range (up to 130 °C), maintaining a continuous increase during heating [25]. These superior water dynamics are attributed to the filler modifying water towards a more thermally stable, “bound” configuration, which resists evaporation and maintains proton transport pathways. In terms of proton conductivity, N-sSLM5 exhibits the highest conductivity (e.g., 179.59 mS cm^−1^ at 120 °C, 90% RH). Crucially, it maintains remarkably high conductivity even at very low relative humidity (30.24 mS cm^−1^ at 120 °C, 20% RH), conditions under which pristine Nafion largely ceases to conduct [26]. This is attributed to the formation of stable “bound” water and the inherent proton-conducting properties of sSLMs. Thermomechanical stability is also significantly enhanced. sSLM incorporation increases the storage modulus and shifts the glass transition temperature (Tg) to higher values (e.g., 180 °C for sSLM-filled Nafion vs. 120 °C for recast Nafion), indicating restricted polymer chain mobility due to strong interfacial interactions.

Similarly to sSLM in Nafion, SSLM has also been successfully applied to the sPEEK matrix [26]. The incorporation of SSLM leads to the formation of a “nacre-like structure,” which is a powerful biomimetic principle applied to synthetic membranes. This highly organized, layered arrangement of stiff nanofillers within a softer polymer matrix enables efficient stress transfer and crack deflection mechanisms, imparting exceptional toughness and strength. This structure significantly increases the storage modulus (e.g., 260 MPa for sPEEK5-SSLM 5 wt%, 3.5-fold higher than pristine sPEEK5) and tensile strength (68.32 MPa, a 160% improvement). The glass transition temperature is also shifted upward by approximately 40 °C.

Despite the hydrophilic nature of SSLM particles, water uptake can decline with increasing SSLM content (e.g., halved at 5 wt% SSLM for sPEEK5), indicating restricted polymer chain mobility and reduced water channel volume. However, water diffusivity at high temperatures is improved, with mobile water retained under dehydrating conditions. The high number of organo-sulfonic functionalities increases the IEC. SSLM platelets provide physical crosslinking, generating continuous proton migration networks, which are crucial for the Grotthuss mechanism in dehydrating conditions. Proton conductivity significantly exceeds pristine sPEEK (e.g., 12.8 mS cm^−1^ at 90 °C, 30% RH for sPEEK5/SSLM 5%, which is 10 times higher than sPEEK and almost 2 times higher than Nafion). Improved hydrolytic stability is also observed. This tailored functionalization of silica highlights the concept of designer nanofillers where the surface chemistry of the filler is specifically tuned to create optimal interfacial compatibility and electrostatic interactions with the chosen polymer matrix. It is not just about adding a hydrophilic site, but about ensuring that the filler’s functional groups complement or physically crosslink with the polymer’s functional groups. This leads to a polymer-filler-specific synergy that dictates the ultimate performance, emphasizing that a “one-size-fits-all” filler approach is inefficient and that rational design based on polymer chemistry is key.

- Similarly, **PSLM (Phosphonated SLM)** was synthesized from 3-(trihydroxysilyl) propyl methyl phosphonate, monosodium salt, yielding a layered material with phosphonate functional groups [24]. This material has shown significant improvements in mechanical strength, water retention, and proton transport when incorporated into sPEEK nanocomposite membranes. PSLM increases the storage modulus of sPEEK and extends its thermal resistance (sPEEK-PSLM3 remains stable up to 200 °C, with a Tg at 245 °C) [26]. In terms of water retention, PSLM helps sPEEK retain water above 60–80 °C, unlike pristine sPEEK which rapidly dehydrates. T_1_ analysis reveals that water molecules are distributed between the polymer and filler acid sites, with strong interactions slowing evaporation. For proton transport, PSLM creates an appropriate network that promotes efficient Grotthuss-type proton transport via highly connected paths. sPEEK-PSLM3 achieves conductivity values close to Nafion 212, especially at low hydration levels (20–30% RH). Furthermore, PSLM increases the chemical resistance of the membrane, preventing sPEEK backbone degradation.

### 3.4. Other Hybrid and Blended Approaches for Fuel Cell Applications

**Figure 5:**
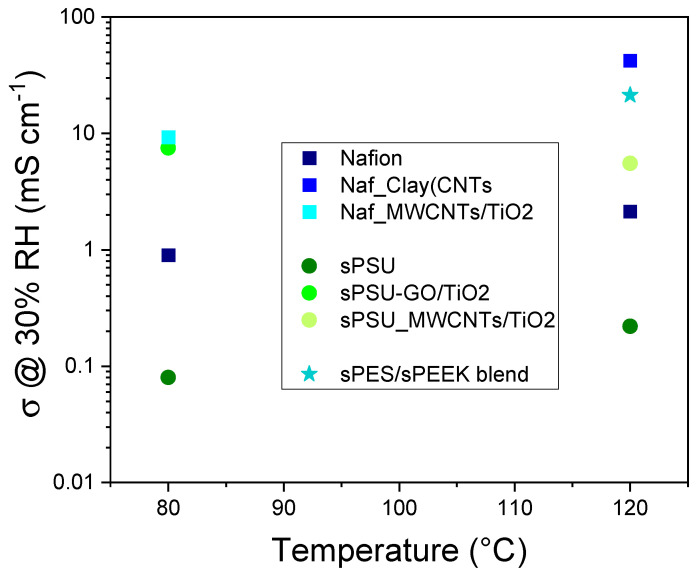
Performance comparison in terms of proton conductivity for blended PEMs and nanocomposite membranes comprising hybrid fillers. Polymer matrices: Nafion and sPSU. For all the systems, the values at optimal filler loading are reported. IEC Nafion: 0.94 meq/g, IEC sPSU: 1.39 meq/g.

Beyond these primary nanocomposite strategies, the PCAM Lab has also explored other hybrid and blended approaches that have shown significant promise in addressing the complex challenges of fuel cell membranes. In this regard, Figure 5 summarizes the peak conductivity performance achieved with the latter approach.

- **Branched Clay-CNT Hybrids**: A new class of hybrid materials based on carbon nanotubes (CNT) rooted on smectite clays (SWy) is synthesized by catalytic chemical vapor deposition (CCVD) [27]. The CNTs are subsequently oxidized and organo-functionalized with hydrophilic groups, such as -RSO_3_H. This process creates a “branched structure” that combines the 2D geometry of clay with the 1D nature of CNTs. This “branched structure” is a sophisticated design that addresses the dual, often conflicting, requirements of PEMs: high proton conductivity and low fuel permeability. The 2D/1D hybrid acts as a multi-scale physical barrier, increasing tortuosity for larger methanol molecules while simultaneously offering an efficient, interconnected network of acid sites for proton hopping. This is a highly advanced strategy for decoupling the transport of desired (protons) and undesired (methanol) species, crucial for DMFCs [28]. These materials guarantee very high proton diffusion even in “quasi-anhydrous” conditions, ensuring proton mobility via a network formed by long, functionalized nanotubes distributed through the clay nanoplatelets. Nafion composites with SWy-oxCNT-RSO3H show proton conductivities of 7 × 10^−2^ Scm^−1^ at 120 °C and 30% RH, which is an order of magnitude higher than pristine Nafion. The branched structure effectively obstructs methanol diffusion, leading to reduced methanol crossover. DMFC tests confirm reduced methanol crossover while maintaining appropriate proton conductivity, especially at low humidity and high temperature (above 100 °C).
- **GO-TiO_2_**: This is a nanostructured hybrid material comprising TiO_2_ nanoparticles grown and stabilized on graphene oxide (GO) platelets [29], which was synthesized via a new, simple, one-pot hydrothermal procedure. This hybrid ensures homogeneous dispersion and prevents the agglomeration of TiO_2_ nanoparticles within the polymer matrix [30,31]. The addition of GO-TiO_2_ to sPSU produces a highly stable network, leading to a three-fold increase in the storage modulus compared to filler-free sPSU and shifting Tg from approximately 200 °C to ~240 °C. GO-TiO_2_ composites demonstrate very high water-retention capacity at elevated temperatures and remarkable proton mobility, particularly in very low relative humidity conditions [30], with proton conductivity two-fold higher than Nafion at 90 °C and RH 20%.
- **MWCNTs-TiO_2_**: The incorporation of this hybrid nanofiller into Nafion (NMT-x) or sPES (PMx) matrices significantly boosts dimensional stability, hydrophilicity, and overall physicochemical properties [32,33]. The synergy between the elongated MWCNTs and the TiO_2_ nanoparticles creates a physically interconnected network at the microscale that retains water and provides extended proton superhighways.

The optimized NMT-3 (Nafion composite) exhibits a plateau in water diffusion up to 130 °C, with diffusivity almost one order of magnitude higher than Nafion recast [32]. This translates into remarkable fuel cell performance, where the NMT-3 achieved a maximum power output of 307.7 mW/cm^2^ at 120 °C and 30% RH, representing a three-fold increase over the Nafion 212 benchmark.

- **Blending approach:** Blended electrolyte membranes based on sulfonated polyethersulfone (sPES) and sulfonated polyetheretherketone (sPEEK), prepared in various ratios (e.g., 50/50 and 25/75) via a simple solution casting process, exhibit enhanced flexibility and thermal resistance without evidence of phase-segregation [34]. Thermogravimetric analysis (TGA) shows higher degradation temperatures and decreased mass loss, indicating improved thermal properties for the blended membranes.

Proton transport is significantly facilitated in these blended membranes, with water diffusivity increasing by at least one order of magnitude in the 50/50 blend. Proton conductivity values are superior to pristine sPES, particularly under lower hydration conditions. Methanol permeability is dramatically reduced by more than 3 orders of magnitude compared to pristine sPES. DMFC tests confirm outstanding performance, with the 25/75 blend reaching a power density of 130 mW cm^−2^ at 80 °C in 4 M methanol solution. Methanol crossover values for the blended membranes are notably lower than Nafion 212, even at high methanol concentrations. Polymer blending is a powerful macroscopic engineering tool that allows for the combination of complementary properties from different polymers without the complexities of nanoscale filler dispersion. The success lies in achieving miscibility, which ensures a uniform distribution of functional groups and structural features from both components. This approach offers a cost-effective and scalable alternative to complex nanocomposites for achieving a balanced set of properties, particularly for DMFCs, where methanol resistance is critical.

The detailed descriptions of LDH, graphene-based, and organosilica nanofillers consistently highlight their ability to simultaneously improve multiple membrane properties (e.g., water retention, mechanical strength, proton conductivity, methanol barrier) through diverse mechanisms (physical crosslinking, creating new pathways, structuring water, increasing tortuosity). This consistent observation emphasizes that the most effective nanocomposite strategies are those that leverage the multifunctionality of fillers. Rational design of advanced PEMs involves selecting or engineering nanofillers that can address several limitations concurrently, leading to synergistic enhancements that are greater than the sum of individual property improvements. This moves beyond simple material addition to a more sophisticated, integrated design approach for complex membrane systems.

The progression in the PCAM Lab’s research from incorporating fillers to developing “sophisticated dual-layer hybrid membranes”, “nacre-like structures,” and “branched structure” Clay-CNT hybrids demonstrates a deliberate manipulation of filler orientation and multi-dimensional assembly. This indicates that the field of PEM development is advancing towards architectural engineering at the nanoscale and microscale. This involves not just the choice of materials but also their precise arrangement and orientation within the polymer matrix to create optimized, often anisotropic, transport pathways and selective barriers. This level of control allows for the decoupling of desired (proton) and undesired (methanol) transport, leading to highly tailored and high-performing membranes for specific fuel cell applications as summarized in Table 1.

## 4. Foundational Contributions: Sulfonated Polysulfone (sPSU)—Architectural Constraints and Trade-Offs

Sulfonated Polysulfone (sPSU) forms the bedrock of much of the PCAM Lab’s research, and a deep understanding of its structure-performance relationship, achieved through multi-scale analysis, reveals critical manufacturing-induced constraints.

### 4.1. Advantages of sPSU as a Promising PEM Material

Within the PCAM Lab’s research, Sulfonated Polysulfone (sPSU) has been increasingly recognized as a compelling and concrete alternative to Nafion for the development of proton exchange electrolytic membranes [35]. Its appeal stems from several key advantages: large market availability, low environmental impact, excellent film-forming ability, and remarkable thermo-mechanical resistance. These attributes are coupled with interesting proton conductive properties. sPSU can be synthesized from commercially available polysulfone, a polymer well-known for its inherent chemical, thermo-oxidative, and thermal stability. The properties of sPSU membranes, particularly their proton conductivity, are closely linked to the degree of sulfonation (DS) [36]. While a high DS can significantly increase proton conductivity, it often leads to undesirable side effects such as excessive swelling and reduced mechanical strength. This necessitates a careful balance between achieving adequate ion transport properties and preserving the chemical and mechanical stability of the polymer material.

### 4.2. Elucidating Structure-Performance Relationships of sPSU

A combination of computational and experimental techniques was applied to highly sulfonated sPSU (DS = 80%) to map its molecular architecture [37,38]. Molecular Dynamics (MD) simulations revealed a defining microstructure: an interconnected lamellar-like structure characterized by ionic clusters approximately 14–18 Å in diameter. These clusters correspond to the hydrophilic sulfonic-acid-containing phase. These clusters represent the hydrophilic, sulfonic-acid-containing phase, and their architecture dictates the overall proton transport efficiency. This computational modeling provided a fundamental explanation for sPSU’s observed properties, moving material development beyond empirical trial-and-error.

Experimental validation using ^1^H Pulsed Field Gradient (PFG) NMR spectroscopy confirmed the mobility of water within the membrane, estimating a hydration number of about 8 mol H_2_O/mol SO_3_^−^ at 80 °C [38] at 80 °C, aligning closely with theoretical simulations. While pristine sPSU exhibits lower dimensional stability and proton conductivity compared to Nafion, its unique microstructure and stability features provided a promising starting point for nanocomposite enhancement [39]. Nonetheless, this systematic approach, which explicitly integrates computational methods with a range of experimental characterization techniques, is crucial for rational material design in complex systems like PEMs. It allows researchers to move beyond empirical trial and error by providing a deep, mechanistic understanding of structure-performance relationships, enabling targeted engineering of materials at the molecular and nanoscale to achieve desired macroscopic properties, thereby accelerating the development cycle and increasing the predictability of new material performance.

### 4.3. Impact of Manufacturing Processes (Recast vs. Mechanical Extrusion) on sPSU’s Anisotropic Behavior

The method of membrane manufacturing, often considered merely a scale-up step, was shown to fundamentally dictate the final morphological, mechanical, and transport characteristics of sPSU films. A critical comparative study analyzed solution casting (recast) versus mechanical extrusion [39].

Mechanical extrusion induces a preferential orientation of polymer chains, revealed by micrometer-sized cleavage planes oriented parallel to the membrane surface in the cross-section. This preferential alignment yields significant improvements in mechanical durability and anti-swelling ability. Extruded sPSU exhibited an outstanding anti-swelling capability and dramatically improved tensile strength (42.3 MPa) compared to recast sPSU (26.5 MPa). The improved strength is crucial, potentially allowing for the production of thinner, mechanically resistant membranes, which reduces internal resistance and boosts cell performance. However, this mechanical gain introduces a critical performance constraint: proton anisotropy. The polymer chain alignment partially hinders proton transport in the direction perpendicular to the membrane surface (through-plane). Consequently, the in-plane conductivity parallel to the extrusion direction (σIP//) is significantly higher than (up to 1.6 times higher at 120 °C) than the crucial through-plane conductivity (σTP). For practical PEMFC applications, isotropic or through-plane enhanced conductivity is paramount. This observation reveals a profound trade-off: mechanical durability, enhanced by chain alignment, paradoxically impedes the desired proton flow across the membrane thickness. This necessitates a process-material co-design approach in future work to ensure that manufacturing techniques preserve mechanical advantages while simultaneously overcoming the detrimental anisotropy in through-plane conductivity.

Table 2 summarizes the properties of sPSU-based membranes, demonstrating the trade-offs between mechanical strength and transport properties achieved through different manufacturing methods and nanocomposite strategies. As is possible to see, our multi-scale investigation of sPSU reveals that the specific molecular architecture (lamellar-like, ionic cluster size) dictates the water’s behavior at the nanoscale (dynamics, hydration number), which in turn profoundly influences the bulk physico-chemical and electrochemical performance. The manufacturing process, by altering polymer chain and ionic cluster orientation, creates a critical trade-off: improved mechanical robustness (allowing thinner membranes) paradoxically impedes the desired proton flow across the membrane thickness. This highlights that for real-world PEMFCs, isotropic or through-plane enhanced conductivity is paramount, suggesting that while extrusion offers mechanical advantages, its current implementation for sPSU may require further optimization to re-orient ionic pathways or be combined with other strategies (e.g., fillers) that can overcome this anisotropy.

## 5. State of the Art in Proton Exchange Membranes: Beyond PCAM Lab’s Specific Contributions

To validate the significance of the PCAM Lab’s work, a direct performance comparison with similar advanced PEMs systems reported in leading literature is essential.

### 5.1. Current Commercial Landscape and Dominance of PFSA Membranes

Nafion, a perfluorosulfonic acid (PFSA) membrane, has historically been the industry standard for PEMs due to its excellent proton conductivity and chemical stability under fully hydrated, moderate temperature conditions [40]. It remains the most widely distributed PEM material globally [41] and continues to dominate the market, particularly for low-temperature applications [42]. Its established supply chain, backed by manufacturers like Chemours, and its proven reliability contribute significantly to its strong market position [43]. However, as discussed, Nafion’s limitations at high temperatures and low humidity, its high methanol crossover rate, its considerable cost, and the environmental concerns associated with PFAS formation during its production and disposal are significant drivers for the ongoing research into alternative materials.

In addition to Nafion, which maintains a strong foothold, other commercial PFSA membranes are available. These include Aquivion (SSC-PFSA) by Solvay, Flemion by Asahi Glass Engineering, Fumion by Fumatech, Aciplex-S by Asahi Kasei, Dow Chemicals (XUS), and GORE-SELECT by Gore [40]. Aquivion, for instance, with its shorter side chains compared to Nafion’s long-side chain PFSA (LSC-PFSA), offers distinct advantages. These include higher crystallinity and a higher glass transition temperature (Tg ca. 140 °C for Aquivion vs. 100 °C for Nafion), which enable improved performance at lower relative humidity and higher temperatures (up to 110 °C) [44].

The global hydrogen fuel cells market is experiencing robust growth, with PEMFCs commanding a substantial share. In 2024, PEMFCs accounted for 33.1% of the market share by technology [44] and approximately 90% by product type [45]. The global PEMFC market size, valued at USD 1.56 billion in 2020, is projected to reach USD 22.74 billion by 2028, exhibiting an impressive Compound Annual Growth Rate (CAGR) of 40.6%. This growth is driven by increasing demand for clean energy, advancements in fuel cell technology, and supportive government policies promoting hydrogen infrastructure.

### 5.2. Emerging Non-Fluorinated and Hydrocarbon-Based PEMs

The imperative for cost-effective and environmentally friendly alternatives to Nafion has spurred extensive research into non-fluorinated and hydrocarbon-based PEMs.

As mentioned above, sulfonated aromatic polymers have emerged as promising candidates due to their chemical tunability, environmental compatibility, and cost-effectiveness [46]. These include sulfonated polyether ether ketone (sPEEK), sulfonated polyimide (SPI), sulfonated polyether sulfone (sPES), sulfonated polyphenylsulfone (sPPSU), and sulfonated poly (aryl ether nitrile) (SPEN) [6].

- **sPEEK**: This material is considered a promising alternative to perfluorosulfonic acid membranes due to its excellent thermal stability, mechanical properties, and tunable proton conductivity [47]. Its properties can be controlled by adjusting the degree of sulfonation (DS), which influences hydrophilicity and proton conductivity. However, a high DS can lead to excessive water uptake, resulting in an extremely high swelling ratio and deterioration of mechanical and chemical stability, which limits its commercial application. To address these issues, various composite membranes are developed by combining sPEEK with a range of organic and inorganic materials, enhancing mechanical and chemical stability, reducing fuel permeability, and improving overall performance [48].
- **SPI**: Sulfonated polyimide membranes have demonstrated reasonable mechanical properties and proton conductivity at 80 °C, even after aging at 130 °C [49]. Polymer chain scission primarily occurs in the early stages of aging, but the membranes largely retain their mechanical integrity. Blending SPI with other polymers, such as polyethersulfone (PES), can significantly increase the stability of the entire membrane and restrict swelling, although it may slightly decrease fuel cell performance if the PES content is too high [49].
- **SPEN**: Sulfonated poly (aryl ether nitrile) (SPEN) typically possesses excellent properties, but its performance is highly dependent on the degree of sulfonation [50]. Balancing the DS with conductivity, mechanical properties, methanol permeability, and dimensional stability is crucial. Modified SPEN membranes have shown high proton conductivity (e.g., 0.137–0.174 S·cm^−1^ at 80 °C, which is higher than Nafion 117), excellent selectivity (8.7 times higher than Nafion 117), and good dimensional stability (e.g., 14.22% swelling at 80 °C).

Beside these polymers, **nanocellulose-based PEMs** have emerged as promising alternative options given their renewability, thermal and mechanical stability, low cost, and hydrophilicity [11]. These bio-based materials leverage the anionic nature of most nanocelluloses, as well as their facile modification with conductive functional groups, to endow ionic conductivity. Performance metrics include good thermal-oxidative stability (up to 190 °C), mechanical robustness (Young’s modulus as high as 1.15 GPa and storage moduli >13 GPa), and high moisture-uptake capacity (ca. 6330% after 48 h). Sulfonic acid crosslinking of nanocellulose, for instance, using sulfosuccinic acid, can simultaneously improve mechanical robustness, water stability, and proton conductivity (up to 15 mS/cm in the fully hydrated state at 120 °C) [51]. Furthermore, cellulose nanocrystals (CNC) blends with poly (vinyl sulfonic acid) (PVS) have been shown to form interlayers that effectively suppress the generation of reactive oxygen species, slow the rate of membrane thinning, and significantly improve the durability of PEMFCs [52].

Within the class of nanomaterials, Metal–Organic Frameworks (MOFs) and Covalent Organic Frameworks (COFs) are examples of porous crystalline materials that are gaining attention for their potential in PEM applications.

- **MOFs**: Metal–Organic Frameworks are porous inorganic–organic hybrid materials that have attracted extensive attention in gas storage, gas separation, and reaction catalysis. When incorporated into polymer matrices, MOFs enhance the proton transfer path within the membrane, providing valuable insights into the mechanism of proton transfer in hybrid membranes [6]. They can be immersed with various proton carriers, and their organic ligands can be modified with functional groups to enhance acidity and hydrophilicity, providing more proton conduction sites [53]. MOFs’ large specific surface area allows composite membranes to accommodate more bound water, which improves proton hopping conductivity, and their numerous coordinatively unsaturated metal sites (CUSs) can form hydrogen-bond networks, promoting proton conduction via the Grotthuss mechanism. This incorporation also enhances mechanical strength, chemical stability, and thermal resilience [54].

Despite their potential, MOFs face several challenges. The high manufacturing cost of purely MOF material crystalline membranes restricts their application in fuel cells, and their performance can be unstable [53]. It is also very difficult to directly process MOFs for fuel cells due to their special and diverse crystal structures, making hybridization with other polymers a common strategy [53]. Excessive addition of low conductivity MOF fillers can lead to internal reunion (agglomeration), which may offset their positive influence on ionomer transport properties and cause resistance to conductivity. Furthermore, some MOF-containing membranes still rely excessively on moisture content to maintain stable performance, with performance seriously reduced at low humidity.

- **COFs**: Covalent Organic Frameworks are an emerging class of organic porous crystalline materials composed of organic linkers connected by strong covalent bonds [55]. Their unique characteristics, including well-ordered and tailorable pore channels, permanent porosity, high crystallinity, and excellent chemical and thermal stability, enable COFs to be potential proton conductors in fuel cell devices [55]. COFs display prominent superiorities in constructing rigid ordered proton transfer channels and improving fuel cell performance and long-term durability [56]. Functionalized COFs have achieved proton conductivities exceeding 0.89 S cm^−1^ at 90 °C under 100% relative humidity (RH), comparable to commercial Nafion membranes [57]. When integrated into PEMFC cathodes, COF-modified ionomers have enabled fuel cells to achieve peak power densities 1.6 times higher than those without COF incorporation.

Generally, proton conduction of COFs is dependent on the amount of water (extent of humidity), necessitating complex water management systems for operation around 80 °C. Challenges persist in terms of membrane durability, scalability, and performance under low humidity or high-temperature conditions. The synthesis of stable and acid-resistant host frameworks remains a major challenge.

Finally, **Ionic liquids** (ILs), which are organic salts typically liquid at temperatures lower than 100 °C, characterized by high conductivity and thermal stability [58]. They have seen increased use in middle and high-temperature PEMFCs [59]. Functionalized ionic liquids (FILs) incorporate a variety of ion exchange groups in their structure, which improve and accelerate proton conduction. Protic Ionic Liquids (PILs), which result from combining a Brønsted acid and a base, are particularly suitable for fuel cell applications due to the active proton available at the cation. They offer advantages such as thermal stability, low volatility, and high conductivity, especially above 100 °C [58].

A significant drawback of ILs in PEMs is leaching from the membranes during operation, which can lead to reduced performance. Strategies to mitigate IL leaching include incorporating ILs into a polymer solution, impregnating the polymer with IL, and cross-linking the ILs with the polymer matrix [60]. The presence of covalent bonds between ILs and polymer chains during the cross-linking reaction is an effective method to decrease IL release. Immobilized PILs, such as poly (diallyl dimethyl ammonium trifluoride methane sulphonate) blended with polybenzimidazole (PBI), have shown promise in mitigating leaching while improving proton conductivity [59]. For example, a blend of PBI-I and P showed increased proton conduction from 0.04 S/cm for PBI to 0.07 S/cm at 150 °C.

### 5.3. Advancements in High-Temperature PEMs (HT-PEMFCs)

High-temperature PEMFCs (HT-PEMFCs) operate between 100 °C and 200 °C, offering several benefits over low-temperature PEMFCs. These advantages include improved electrode kinetics, better tolerance to fuel impurities (particularly CO), simplified thermal and water management systems, reduced dependency on cooling systems, and potential for higher overall system efficiencies. The high CO tolerance of anode catalysts in HT-PEMFCs makes it possible to use hydrogen directly from simple methanol reformers, simplifying or removing the need for selective oxidants and CO separators from the processing system. 

Specific material developments for HT-PEMFCs include phosphoric acid (PA)-doped polybenzimidazole (PBI) membranes, which have demonstrated satisfactory proton conductivity under high-temperature and anhydrous conditions [61]. This has led to significant advancements in HT-PEMFC design and development [62]. Novel self-cross-linked, net-structured proton-conducting polymer membranes, such as poly (benzimidazole-co-aniline) (PBIANI), have shown improved mechanical strength (e.g., stress at break of 26 ± 3 MPa for 45 wt.% phosphoric acid doped PBIANI) and enhanced proton conductivity (167 mS cm^−1^ at 120 °C and 100% RH), with proton conductivity showing a marginal effect of humidity [62].

Despite these advancements, the persistent issue of phosphoric acid (PA) leaching remains a significant challenge for HT-PEMFCs. This leaching can lead to a sharp drop in cell output voltage and can cause degradation and corrosion of fuel cell components, further reducing performance and durability. The “free PA” in the membrane, which is the main carrier for proton transfer, is weakly bonded to the polymer by hydrogen bonding, leading to its inevitable leaching during HT-PEMFC operation. Strategies aimed at mitigating PA leaching include designing crosslinked structures, incorporating hygroscopic nanoparticles, and improving the alkalinity of the polymer [62].

### 5.4. Manufacturing Innovations for PEMs

Advancements in manufacturing processes are crucial for translating laboratory-scale PEM innovations into commercially viable products. Although not exhaustive, we provide a brief overview of advanced fabrication techniques:

- **Roll-to-roll (R2R) Coating**: This continuous manufacturing method is considered key for achieving high throughput and scalability, addressing the pressing need for faster, more cost-effective production of Membrane Electrode Assemblies (MEAs) [63]. Techniques like microgravure and slot-die coating are being optimized for applying catalyst layers and fabricating membranes. R2R coating offers significant cost reductions through economies of scale, despite potentially high initial capital costs. Microgravure, a self-metered technique, has achieved platinum loadings comparable to commercial targets for light-duty vehicles. Slot-die coating offers flexibility and precision with proper optimization, although it can have issues with cracking [63].
- **Additive Manufacturing (3D Printing)**: Also known as 3D printing, this technology enables the creation of complex geometries and structures, improving fuel cell performance and efficiency [63]. It offers advantages such as reduced material waste, improved precision, and rapid prototyping. Crucially, additive manufacturing can integrate multiple conventional parts (e.g., liquid/gas diffusion layer, bipolar plate, gasket, and current distributor) into one multifunctional plate, for the first time [64]. This integration eliminates interfacial contact resistances between parts, leading to significantly increased energy efficiency (up to 86.48% at 2 A/cm^2^ and 80 °C) and hydrogen generation rates (increased by 61.81%) compared to conventional designs.
- **Electrospinning**: Nanofiber webs prepared by electrospinning can be used as a reinforcement matrix in PEMs, significantly improving mechanical properties, chemical stability, and durability [65]. The nanofibers can be welded together where they intersect, forming welded joints that make the nanofiber web stronger and stiffer, thereby improving the mechanical strength and hydration stability of the PEM. Alternatively, nanofibers can be impregnated with useful additives, such as inorganic free radical scavengers, that diffuse out slowly, enhancing the chemical stability of the PEM over time.
- **Precision Chemical Machining**: Processes like photochemical etching are utilized to create high-precision components critical for efficient energy conversion in hydrogen fuel cells, allowing for intricate designs and tight tolerances [66]. This innovative process avoids the introduction of mechanical stress or material distortion, preserving the material’s inherent properties and leading to improved durability and increased efficiency of components.

These manufacturing innovations are critical for driving down the capital costs of PEM electrolyzer systems (e.g., over 90% reduction since 2001, from ~$17,500/kW to ~$1300/kW in 2020) and fuel cell stacks [67]. They contribute to improved power density and conversion efficiency, enabling the mass production required for widespread commercial adoption. Efforts are ongoing to further reduce costs in both stacks and the balance of the system to meet ambitious clean hydrogen cost goals.

### 5.5. Commercial Hydrocarbon Benchmark: Pemion^®^

A significant development in the non-fluorinated PEM space is the commercialization of hydrocarbon membranes, such as Pemion^®^ by Ionomr Innovations. This material, based on a sulfo-phenylated polyphenylene (sPPX) backbone, serves as a critical state-of-the-art benchmark for hydrocarbon-based systems developed in the lab. As a reinforced, PFAS-free membrane, it is designed to address the environmental and end-of-life concerns of PFSA ionomers while delivering high performance [68].

Published data highlights its potential for next-generation, high-temperature applications:

- **Electrochemical Performance**: Pemion^®^-based cells demonstrate a significantly reduced detrimental influence of high temperatures compared to PFSA-based cells. At an operation temperature of 110 °C, 250 kPa (abs), and 50% RH, Pemion^®^ achieved a peak power density of 0.96 W cm^−2^, which was 8% higher than a short-side chain PFSA reference cell (0.89 text W cm^−2^. Under H2/air (80% RH, 80 °C, 250 kPa (abs)), it reached a peak power density of 1.1 W cm^−2^, reaching performance comparable to state-of-the-art PFSA systems.
- **Mechanical Properties**: Pemion^®^ is a mechanically reinforced membrane. Technical data sheets report robust tensile properties, with tensile strength values greater than 50 MPa and Young’s Modulus values exceeding 600 MPa [68]. Thermo-mechanical analysis shows that its Young’s modulus and strain hardening are temperature-independent, whereas reinforced PFSA materials exhibit significant decay above 90 °C. This mechanical toughness is attributed to its sterically encumbered, rigid-rod polyphenylene backbone.
- **Durability**: Critically, Pemion^®^ has been validated against industry-standard durability protocols. It successfully met and exceeded established accelerated durability benchmarks for combined chemical and mechanical stress testing. Throughout 1000 h of cyclical testing (intermittent dry and wet conditions under high-voltage chemical stress), Pemion^®^ exceeded the 20,000 cycle durability targets set by the US Department of Energy (US DOE) by more than two-fold. Furthermore, in cross-pressure accelerated mechanical stress tests (ΔP-AMST), reinforced Pemion^®^ membranes demonstrated a longer lifetime than incumbent reinforced PFSA materials.

This commercial benchmark provides a clear set of performance and durability targets that lab-scale materials, such as those from the PCAM Lab, must meet or exceed. A direct comparison (Table 3) reveals the competitive standing of PCAM’s strategies.

## 6. Long-Term Durability, Stability Limits, and Improvement Strategies

### 6.1. Intrinsic Stability Limitations of Aromatic Hydrocarbon PEMs

Durability remains the most significant barrier to the widespread commercialization of fuel cells. The shift from perfluorinated Nafion to cost-effective hydrocarbon membranes introduces critical, intrinsic stability limitations that must be addressed through sophisticated engineering strategies [73]. Currently, the Intrinsic Stability Limitations of Aromatic Hydrocarbon PEMs are as follows:

- **Chemical Degradation via Radical Attack.** The primary failure mode of aromatic hydrocarbon PEMs, such as sPSU and sPEEK, is chemical degradation triggered by Reactive Oxygen Species (ROS), predominantly hydroxyl radicals (•OH) and hydrogen peroxide (H_2_O_2_), which are generated during fuel cell operation. These radicals attack the relatively vulnerable C-H bonds present in the polymer’s aromatic backbone, leading to chain scission, reduced molecular weight, and eventual loss of ionic conductivity and mechanical integrity [74]. Furthermore, the functional groups themselves are susceptible to thermal degradation; sulfonic acid groups in sPEEK membranes are significantly reduced when temperatures exceed ~200 °C [62,75].
- **Mechanical Degradation and Dimensional Instability**: The pursuit of high proton conductivity necessitates a high degree of sulfonation (DS), which directly conflicts with the maintenance of dimensional stability. This is termed the DS-swelling paradox. High DS leads to catastrophic water uptake and excessive swelling, as noted for pristine sPEEK, which can exhibit swelling close to 200% after prolonged treatment [76,77]. This excessive swelling destabilizes the polymer microstructure, causing the collapse of proton transport channels and mechanical weakening, leading to failure modes like cracking and thinning during cyclic operation, load cycling, or temperature cycling. Operation at elevated temperatures (≥90 °C) exacerbates these mechanical stresses. This type of degradation occurs due to various operational stressors, including cyclic operation, load cycling, frequent start-stop cycles, low humidification or humidification cycling, and operation at temperatures of 90 °C or higher [73]. These conditions can lead to membrane thinning, cracking, and loss of mechanical integrity [52].

The extensive list of degradation mechanisms clearly demonstrates that PEM durability is not a single material property but a complex interplay of chemical, mechanical, and electrochemical factors, often exacerbated by dynamic operating conditions and interactions with other system components [78]. Solutions are correspondingly multi-pronged, involving material science, MEA design, and operational strategies. Achieving long-term durability for PEMFCs thus requires a holistic, systems-level approach to research and development. It necessitates understanding and mitigating degradation pathways across all components of the MEA and their interfaces, under a wide range of realistic operating conditions. This underscores the need for advanced in situ and operando characterization techniques to probe complex interfacial chemistries and degradation mechanisms [79].

Furthermore, cost reduction is consistently highlighted as a primary goal in PEM development [73]. However, it is also explicitly stated that cost reduction often involves using “less or cheaper materials,” which can directly “negatively affect durability and lifetime” [80]. This creates critical tension in the commercialization pathway. The pursuit of cost-effective PEMs cannot compromise on durability. Any cost savings from cheaper materials or reduced noble metal loading must be balanced against the potential for reduced operational lifetime or increased maintenance costs. This drives research towards developing inherently stable, low-cost materials (e.g., non-fluorinated polymers, PGM-free catalysts) that can meet both performance and durability targets simultaneously, rather than simply accepting trade-offs.

### 6.2. The Nanocomposite Challenge: Interfacial Stability and Filler Leaching

While nanocomposites are proposed as a solution, one of the most consistent observations in the literature is the non-linear response of proton conductivity to filler concentration. This relationship typically follows a bell-shaped or “volcano” curve, as detailed in Table 4.

Furthermore, the nanocomposite approach introduces an additional, critical durability challenge: nanofiller leaching. This is a primary concern for nanocomposite PEMs. In many systems, the hydrophilic nanofillers (e.g., LDH, sGO, silica) are incorporated into the polymer matrix via non-covalent forces, such as hydrogen bonding or electrostatic interactions. During prolonged fuel cell operation, the constant flux of water through the membrane, combined with swelling/deswelling stresses, can cause these weakly bound fillers to leach out of the membrane. This leaching has two catastrophic consequences:

1. A progressive loss of the very functions the filler was added to provide (e.g., water retention, mechanical reinforcement, proton pathways).
2. The leached filler material can travel to the catalyst layers, poisoning the platinum catalyst and irreversibly degrading cell performance.

Therefore, a key objective in advanced nanocomposite design is not just the initial dispersion of fillers but ensuring their long-term immobilization within the matrix.

### 6.3. Quantifying Durability: Accelerated Stress Test (AST) Protocols

To bridge the gap between lab performance and commercial viability, durability must be quantified using standardized Accelerated Stress Tests (ASTs). These protocols are designed to simulate thousands of hours of real-world operation in a condensed timeframe.

**Chemical ASTs**: These tests are designed to accelerate ROS generation. A common protocol is the Open-Circuit Voltage (OCV) Hold, often at high temperature and low humidity, which maximizes the creation of •OH radicals and attacks the polymer backbone [81]. Membrane failure is often monitored by measuring fluoride-ion release (for PFSA) or crossover current.

**Mechanical ASTs**: These tests target mechanical failure modes like cracking and thinning. The most common is Relative Humidity (RH) Cycling, where the membrane is subjected to thousands of cycles between wet and dry conditions (e.g., 2 min wet, 2 min dry) [82]. This repeated swelling and shrinking induces mechanical fatigue, leading to crack formation.

**Combined Chemical/Mechanical ASTs**: The most rigorous protocols, defined by bodies such as the U.S. Department of Energy (DOE) and Hydrogen Europe, combine these stressors [83]. For example, the Pemion^®^ membrane was validated using a 1000 h test that combined RH cycling with high-voltage chemical stress, ultimately exceeding the 20,000-cycle durability target by more than two-fold. Another advanced test, the ΔP-AMST, combines RH cycling with a constant pressure differential across the membrane to simulate the mechanical stresses in a fuel cell stack.

### 6.4. PCAM Lab’s Targeted Mitigation Strategies

**Physical Crosslinking and Dimensional Restraint**: Nanofillers are deployed specifically to counteract the DS-swelling paradox. Materials like Layered Double Hydroxides (LDH) and Phosphonated Organosilica Layered Materials (PSLM) act as effective physical crosslinkers. By restricting polymer chain mobility through strong electrostatic interactions, these fillers significantly enhance dimensional stability, as evidenced by the 25 °C increase in Tg for sPSU/LDH. This physical crosslinking maintains the stability of the hydrophilic channels, preventing their collapse even under dehydrating conditions.

**Biomimetic Reinforcement**: The use of Sulfonated Siliceous Layered Materials (SSLM) in sPEEK demonstrated the success of architectural engineering in improving mechanical stability. The resulting “nacre-like structure” is a biomimetic principle enabling efficient stress transfer and crack deflection, leading to a 160% improvement in tensile strength. This high mechanical integrity directly mitigates the risk of mechanical degradation (cracking and thinning) associated with hydrocarbon PEMs during dynamic operation.

**Chemical Protection and Scavenging Functionality**: Although primarily cited for their hygroscopic nature, inorganic fillers such as TiO_2_ (used in GO-TiO_2_ and MWCNTs-TiO_2_ hybrids) possess inherent radical scavenging capabilities. By incorporating these materials, the membranes gain a degree of internal chemical protection, although the primary degradation pathway (ROS attack on the C-H bonds) remains an active area of investigation.

### 6.5. Broader State-of-the-Art Improvement Strategies for Non-Nafion PEMs

Beyond PCAM’s specific material focus, broader strategies are employed across the field to enhance hydrocarbon PEM durability:

**Reinforcement Scaffolds and Interlayers**: Utilizing internal reinforcement networks, such as electrospun nanofibers, significantly boosts the mechanical properties, chemical stability, and hydration stability of PEMs. Alternatively, implementing protective gas barrier interlayers, such as nanocellulose-based blends, has been shown to effectively suppress the generation of ROS, thus slowing the rate of chemical degradation and membrane thinning.

**High-Temperature Ionomer Design**: For High-Temperature PEMFCs (HT-PEMFCs, 100 °C to 200 °C), alternative ionomers like phosphoric acid (PA)-doped Polybenzimidazole (PBI) membranes have been developed. These systems operate under anhydrous conditions, removing the dehydration constraint, and exhibit improved tolerance to fuel impurities like CO. However, this strategy introduces a new durability challenge: the persistent leaching of phosphoric acid from the membrane, which degrades cell performance and components.

**Navigating the Cost-Durability Trade-Off**: Achieving industrial viability requires solving the commercial tension between cost and durability. Hydrocarbon membranes are intrinsically lower cost than Nafion. However, successfully integrating the required complex engineering, such as the nanoscale layering and tailored functionalization seen in PCAM’s work, adds complexity and cost to the synthesis and manufacturing process. The research community is consistently faced with the challenge that pursuing cost-effective PEMs by using less or cheaper materials often negatively affects long-term durability and operational lifetime. Therefore, the metric for success is developing inherently stable, low-cost materials that meet both performance and durability targets simultaneously.

## 7. Key Characterization Insights and Methodologies

For the PCAM Lab, comprehensive characterization has been fundamental to understanding the complex interplay between material structure, molecular dynamics, and macroscopic performance in advanced PEMs. A systematic, multi-scale approach, involving computational modeling, spectroscopy, and microscopy, is critical for deepening fundamental understanding and accelerating rational material design. This approach forms a powerful validation loop where findings at one scale are validated and explained by observations at another, which in turn explains macroscopic performance.

### 7.1. Nuclear Magnetic Resonance Spectroscopy

Nuclear Magnetic Resonance (NMR) spectroscopy is a versatile tool for probing molecular dynamics and interactions within PEMs.

- PFG-NMR (Pulsed Field Gradient Nuclear Magnetic Resonance): This technique measures the long-range translational mobility (diffusion coefficient, D) of water, methanol, and ions (e.g., H^+^, Li^+^) within the hydrophilic domains of the membrane. It quantifies molecular mobility, helps distinguish between bulk and bound water populations, and reveals how fillers modify water networks and ion pathways. For example, sPSU/LDH membranes exhibited a water diffusion coefficient of 6.63 × 10^−6^ cm^2^s^−1^ at 130 °C, which is more than ten times higher than that of pristine sPSU. Similarly, Nafion N-sSLM5 composites showed the highest water self-diffusion coefficients across the entire temperature range up to 130 °C.
- T_1_/T_2_ Relaxometry: T_1_ and T_2_ relaxation times provide information about short-range molecular motions and the strength of interactions between molecules and the polymer matrix. This technique reveals local mobility, the state of water (bound vs. free), and its resistance to evaporation. For instance, the T_1_ values for sPSU/LDH consistently increased with temperature, indicating stable water structuring within the composite.
- Spectral Analysis (^1^H, ^13^C): This provides insights into the chemical environment of various species, changes in functional groups, and molecular interactions. It helps identify different water populations and chemical shifts resulting from acidity or specific interactions within the membrane.
- Rheo-MRI (Rheology-Magnetic Resonance Imaging) is a specialized technique used to investigate the organization of 2D nanoparticles within polymer solutions under shear forces. It provides direct visualization of filler alignment induced by mechanical forces during processing. For example, studies on Nafion/LDH composites using Rheo-MRI demonstrated that shearing induced a preferential orientation of LDH lamellae parallel to the shear direction.

The importance of “bound water” beyond simple water uptake is a recurring theme in the PCAM Lab’s research. While total water uptake might not always directly correlate with proton conductivity or durability, the ability of a membrane to retain “bound water” at high temperatures is crucial for sustained performance. NMR relaxation times (T_1_) and spectral analysis are key techniques for distinguishing between bulk and bound water populations. This highlights that it is not just the quantity of water, but its state and interaction with the membrane, that matters for high-temperature performance. Fillers that can strongly bind and structure water molecules (e.g., through strong electrostatic interactions with functional groups) effectively create an “internal humidification system” that resists evaporation and maintains proton mobility via the Grotthuss mechanism, even when “free” or “bulk” water is scarce. This emphasizes the importance of interfacial chemistry between filler and water/polymer in designing robust PEMs.

### 7.2. Electrochemical Impedance Spectroscopy (EIS)

EIS is a fundamental electrochemical technique used to quantify proton/ion conductivity and cell resistance. It is widely employed for assessing the overall fuel cell performance in single-cell tests and can reveal transport anisotropy within the membrane. For example, EIS measurements showed that sPSU/LDH achieved a proton conductivity of 4 mS cm^−1^ at 90 °C and 20% RH, a 20-fold increase over pristine sPSU. Nafion N-sSLM5 demonstrated a conductivity of 30.24 mS cm^−1^ at 120 °C and 20% RH, indicating robust performance under low humidity.

### 7.3. Dynamic Mechanical Analysis (DMA)

DMA measures the viscoelastic properties of materials, including the storage modulus (E’), loss modulus (E”), and tan δ. These parameters provide information about mechanical strength, stiffness, flexibility, and glass transition temperatures (Tg). DMA is crucial for assessing durability, dimensional stability, filler reinforcement effects, and extension of thermal stability in PEMs. For instance, the incorporation of LDH into sPSU increased the Tg from 200 °C for pristine sPSU to 225 °C for sPSU/LDH. In sPEEK5/SSLM 5%, a 160% increase in tensile strength was observed.

### 7.4. Microscopy Techniques

Microscopy techniques provide visual and structural information at various scales.

- **SEM (Scanning Electron Microscopy):** SEM is used to examine the surface morphology, cross-sectional structure, homogeneity, and filler dispersion at micro-to-nanoscale. It provides visual evidence of the material’s architecture and confirms the dispersion state of fillers. For instance, SEM revealed that recast sPSU membranes exhibit a dense, homogeneous structure, while mechanically extruded sPSU membranes show micrometer-sized cleavage planes oriented parallel to the surface.
- **TEM (Transmission Electron Microscopy):** TEM offers higher resolution images of nanoparticle morphology, size, dispersion, and the extent of exfoliation or agglomeration within the polymer matrix. It confirms the nanoscale structure and distribution of fillers. For example, TEM images confirmed the direct growth of TiO_2_ nanoparticles on MWCNTs in MWCNTs-TiO_2_ composites.
- **AFM (Atomic Force Microscopy):** AFM provides information on surface morphology, roughness, and nanoscale phase separation (e.g., hydrophobic/hydrophilic domains). It reveals local structural changes induced by fillers and the distribution of domain sizes.

Computational methods provide theoretical foundations and predictive capabilities, complementing experimental observations.

- MD (Molecular Dynamics) & DFT (Density Functional Theory): These techniques are used to model molecular architecture, estimate structural parameters, and understand interactions within the polymer and with water molecules at a fundamental level. They provide theoretical insights into molecular-level mechanisms. For sPSU, MD simulations revealed an interconnected lamellar-like structure with ionic clusters of 14–18 Å, offering a fundamental explanation for its observed properties.

## 8. Critical Assessment of Commercialization Barriers and Long-Term Feasibility

A critical assessment of the proposed materials requires moving beyond laboratory performance metrics to address the practical constraints of industrial scaling, cost barriers, and long-term durability under realistic operational conditions.

### 8.1. Economic and Market Constraints: Cost vs. Complexity Trade-Off

The primary economic appeal of hydrocarbon membranes lies in the intrinsically lower raw material cost compared to perfluorinated Nafion monomers, whose production costs are significant. This foundational cost reduction provides a compelling case for alternatives like sPSU [84]. However, this raw material advantage is partially offset by the complexity cost barrier introduced by the required nanocomposite engineering. In this regard, the synthesis and functionalization of high-performance designer nanofillers, such as organosilica layered materials (sSLM or PSLM) or the fabrication of specialized MWCNT-TiO_2_ hybrids, benefits of low complexity and energy consumption to the production phase.

The ultimate economic viability is therefore determined by long-term cost amortization. The demonstrated ability of PCAM to operate efficiently at higher temperatures and lower humidity simplifies the fuel cell system architecture by reducing the need for complex and costly external humidification and cooling systems (Balance-of-Plant costs). This long-term operational saving, coupled with higher efficiency (less fuel consumption over product lifetime), must be quantified through detailed economic modeling to prove the net commercial advantage over the costly but reliable Nafion standard.

### 8.2. Manufacturing and Scalability Challenges of Nanocomposites

Translating lab-scale success to high-volume, low-cost commercial production hinges on overcoming significant manufacturing and scalability hurdles inherent to nanocomposite processing. The foremost challenge is maintaining the homogeneous dispersion of nanoscale additives at industrial volumes [85]. Fillers like LDH are hydrophilic, and their incorporation into typically hydrophobic polymer matrices requires complex surface modification or processing adjustments to prevent agglomeration. Agglomeration leads to membrane inhomogeneity, macroscopic defects, and a severe deterioration of both physicochemical and electrochemical performance [86]. Furthermore, achieving the precise structural control demonstrated in the lab, such as the deliberate alignment of 2D layered materials (e.g., LDH for dual-layer membranes) or the creation of the nacre-like structure in sPEEK/SSLM, is exceedingly difficult to replicate reliably during continuous, high-throughput manufacturing processes such as Roll-to-Roll (R2R) coating [87,88]. While R2R is critical for achieving the necessary economies of scale, maintaining nanoscopic architectural precision at high processing speeds remains a defining engineering challenge.

A crucial limitation highlighted by the PCAM Lab’s research on sPSU is the manufacturing-induced anisotropy, which clearly represents an extrusion paradox. Mechanical extrusion, a highly scalable process often desired for commercial membrane production, induces a preferential alignment of polymer chains and ionic clusters. This alignment yields significant mechanical benefits, increasing tensile strength from 26.5 MPa (recast sPSU) to 42.3 MPa (extruded sPSU), which is vital for thin, mechanically resistant membranes. However, this mechanical gain introduces a critical performance constraint: the chain alignment partially hinders proton transport in the through-plane direction (σTP), which is the essential conduction pathway for fuel cell operation. The in-plane conductivity (σIP) can be up to 1.6 times higher than the critical σTP at 120 °C. This observation reveals a profound trade-off: the manufacturing process designed to improve durability paradoxically impedes the desired charge transport. Future process co-design must prioritize overcoming this detrimental anisotropy by engineering fillers to re-orient ionic pathways orthogonal to the shear direction during R2R production.

### 8.3. The Durability Imperative: Transition to Accelerated Stress Testing (ASTs)

Durability remains the most significant barrier to commercialization. For hydrocarbon PEMs, failure is typically complex, involving chemical degradation (ROS attack on C-H bonds) and mechanical degradation (thinning and cracking exacerbated by excessive swelling/deswelling cycles) [89]. The mitigation strategies developed by the PCAM Lab—such as physical crosslinking by LDH and biomimetic reinforcement by SSLM—directly target these intrinsic material weaknesses. However, the operational viability of these solutions must be quantified under dynamic, real-world conditions that mimic actual vehicle drive cycles or start-stop events, which are known to accelerate degradation [90]. To bridge the gap between lab-scale testing and commercial standards, the assessment must incorporate rigorous Accelerated Stress Tests (ASTs), moving beyond static, steady-state performance evaluation. Relevant AST protocols, often defined by bodies such as the U.S. Department of Energy (DOE), include the following:

**Dynamic Load Cycling**: Tests simulating vehicle drive cycles to assess mechanical stress and fatigue on the Membrane Electrode Assembly (MEA).

**Humidity/Temperature Cycling**: Repeated adsorption–desorption cycles designed to induce mechanical failure (cracking, thinning) by stressing the polymer matrix [91].

**Chemical Degradation Monitoring**: Continuous monitoring of degradation product release, such as the F^−^ release used for Nafion, or equivalent protocols for non-fluorine membranes, measured at least every 24 h [92].

**Performance Benchmarks**: Continuous monitoring of key metrics, including High-Frequency Resistance (HFR) and hydrogen crossover, with a target crossover current of less than 20 mA/cm^2^.

Quantifying lifetime under these dynamic AST protocols is the necessary next step to transition the technology readiness level of these advanced nanocomposites toward commercial adoption. Table 5 summarizes the key commercialization hurdles related to the advanced PCAM.

## 9. Holistic Environmental Impact: Life Cycle Assessment (LCA) Perspective

A detailed environmental analysis requires a holistic Life Cycle Assessment (LCA) perspective, comparing the fluorine-free alternatives not just at the material level, but across the full life cycle from raw material extraction and synthesis (production phase) to fuel cell operation (use phase) and eventual disposal (end-of-life) [93].

### 9.1. The PFAS-Free Advantage

The primary environmental benefit of moving to hydrocarbon polymers (sPSU, sPEEK) is the fundamental elimination of per- and polyfluoroalkyl substances (PFASs). Nafion’s fluorine content results in extreme environmental persistence and bioaccumulation, raising concerns over the disposal of used membranes as they are potential sources of toxic perfluorocarboxylic acids (PFCAs) [72,84]. By contrast, non-fluorinated alternatives offer a significant, intrinsic environmental advantage at the end-of-life phase.

### 9.2. Production Hotspots and Environmental Trade-Offs

While the end-of-life profile of hydrocarbon PEMs is superior, the environmental assessment of the production phase presents a critical counter-assessment. LCA studies on comparable engineered PFAS-free membranes (such as sulfonated graphene oxide) indicate that the energy consumption during lab-scale manufacture is the main environmental hotspot, often contributing most significantly to the Global Warming Potential (GWP) [72,93]. The synthesis of complex nanocomposites demands high energy input for processes such as the one-pot sol–gel synthesis of organosilica layered materials or the hydrothermal growth procedures used for GO-TiO_2_ hybrids. Furthermore, the environmental load associated with the solvents (e.g., N-Methyl-2-pyrrolidone, NMP) used in solution casting and the strong acids (H_2_SO_4_) used for sulfonation must be fully accounted for [94]. This leads to a crucial environmental trade-off: the production phase of complex, high-performance nanocomposites may result in a higher initial environmental footprint (higher GWP at lab scale) compared to the proxy for Nafion production [72]. However, this must be amortized by the substantial use-phase benefits.

### 9.3. Amortization of Environmental Impact Through Operational Efficiency

The true environmental superiority of advanced PEMs is established by modeling their performance during operation. The superior proton conductivity and enhanced durability achieved by PCAM enable significant operational efficiencies. For example, LCA analysis shows that better proton conductivity and selectivity can potentially offset the higher impact in the production phase by reducing hydrogen consumption (or methanol fuel consumption) during the use phase of the PEMFC. Materials demonstrating superior performance at high T/RH (e.g., sPSU/LDH and Nafion-sGOsulf) improve the overall fuel cell efficiency, requiring less energy input per kilowatt-hour generated over the cell’s lifetime. The scaling factor is also vital. LCA results consistently demonstrate that the environmental impact is highly scale-dependent, and the GWP contribution can be drastically reduced when laboratory procedures are successfully translated to mass manufacturing, often requiring substantial reduction (e.g., 90%) in electricity consumption per unit of membrane produced to make the environmental impact comparable to the Nafion benchmark. Therefore, future work must couple the performance data with rigorous, scaled-up LCA to validate the overall environmental advantage of these advanced, non-fluorinated materials.

## 10. Conclusions and Future Outlook

### 10.1. Summary of Significant Advancements

The PCAM Lab’s research provides substantial evidence that aromatic hydrocarbon ionomers, specifically sulfonated polysulfone (sPSU) and sulfonated polyether ether ketone (sPEEK), are highly viable alternatives to Nafion. The lab’s evolutionary strategy, which centers on multifunctionality and nanoscale architectural control, has successfully addressed the intrinsic limitations of these materials, particularly their vulnerability to dehydration, excessive swelling, and poor methanol selectivity. Key achievements include the use of Layered Double Hydroxides (LDH) as effective physical crosslinkers to achieve superior dimensional stability and robust high-temperature performance (e.g., 150 mW cm^−2^ in high-concentration DMFCs). Furthermore, the design of proton superhighways using TiO_2_-decorated MWCNTs has enabled record high-temperature operation for Nafion-based composites, tripling power output over the pristine membrane at 120 °C and 30% RH. Finally, the realization of biomimetic, nacre-like structures in sPEEK/SSLM systems validates the principle that rational material design, where filler surface chemistry and orientation are precisely controlled, can simultaneously optimize both mechanical integrity and transport efficiency. These successes confirm that nanocomposite strategies are effective tools for decoupling traditionally linked material properties, such as proton conductivity and fuel permeability, across a wide range of operating conditions.

### 10.2. Critical Research Gaps and Future Directions

Despite these profound advancements, several critical challenges must be addressed to transition these materials toward widespread commercial application:

- **Durability Quantification and Long-Term Stability**: The most critical future direction involves moving beyond short-term performance metrics to rigorous, long-term durability testing. Comprehensive investigations into the chemical, mechanical, and electrochemical stability of these advanced nanocomposites must be performed under realistic, accelerated load cycling and dynamic operating conditions common in commercial fuel cells [74].
- **Mitigating Manufacturing-Induced Anisotropy:** The finding that mechanical extrusion, while boosting mechanical strength, fundamentally degrades the critical through-plane proton conductivity in sPSU is a major application limit. Future research must focus intensely on optimizing manufacturing processes to simultaneously preserve mechanical gains while eliminating this detrimental anisotropy, potentially by combining extrusion with filler alignment or architectural strategies that re-orient ionic pathways.
- **System Integration and MEA Optimization:** The next crucial step is the effective integration of these novel laboratory-scale membranes into industrial Membrane Electrode Assemblies (MEAs). Research is required to optimize MEA design, ensuring uniform current distribution and mitigating interfacial contact resistances. This will require the implementation of advanced, scalable manufacturing techniques such as roll-to-roll (R2R) coating and additive manufacturing.
- **Fundamental Mechanistic Understanding:** Continued multi-scale computational and experimental studies (MD, PFG-NMR) remain vital to deepen the understanding of ion and water transport mechanisms, particularly within the complex interfaces created by the nanocomposite structure. This detailed mechanistic knowledge is essential for the rational design of even more advanced materials with predictable, industrial-scale performance.
- **New Architectures**: Exploration of novel membrane architectures, such as gradient membranes, porous structures, and advanced dual-layer designs, can further optimize transport pathways and mitigate existing limitations, leading to next-generation PEMs with unprecedented performance.
- **LCA Validation at Scale**: Rigorous, scaled-up Life Cycle Assessment must be conducted to prove that the operational efficiency and end-of-life benefits (PFAS elimination) successfully amortize the production phase’s environmental cost (energy consumption during complex nanofiller synthesis).

By focusing on these system-level challenges, the developed membranes can enable simpler, more cost-effective fuel cell systems, accelerating the commercial adoption necessary for contributing significantly to the global clean energy transition.

## Figures and Tables

**Table 1:** Summary of Key Nanofillers and Their Primary Impacts on Membrane Properties.

Nanofiller Type	Functional Groups/Key Structural Feature	Host Polymer	Primary Impact	Key Mechanism	Representative Performance Metric
Layered Double Hydroxides (LDH)	Layered, Fixed Positive Charge, Anion Exchange	sPSU, Nafion, sPEEK	Water Retention, Dimensional Stability, Proton Conductivity, Methanol Barrier	Physical Crosslinking, Grotthuss Enhancement, Increased Tortuosity	sPSU/LDH: 4 mS cm^−1^ at 90 °C/20% RH (20× sPSU)
Sulfonated Graphene Oxide (sGO)	Sulfonic, 2D Layered	Nafion, sPSU	Water Retention, Proton Conductivity, Methanol Barrier	Strong H-bonding Network, Proton Hopping	Nafion-sGOsulf: 231.9 mS cm^−1^ at 90% RH (81% enhancement)
TiO_2_-decorated Graphene Oxide (GO-TiO_2_)	TiO_2_ Nanoparticles on GO, Hybrid	sPSU	Mechanical Strength, Water Retention, Proton Conductivity, Thermal Stability	Homogeneous Dispersion, Internal Humidification	sPSU_GO-TiO_2_ 3%: 2× Nafion conductivity at RH 20%
TiO_2_-decorated Multi-Walled Carbon Nanotubes (MWCNTs-TiO_2_)	TiO_2_ Nanoparticles on MWCNTs, Hybrid	Nafion, sPES	Dimensional Stability, Water Retention, Proton Conductivity	Interconnected Network, Direct Proton Contribution	Nafion NMT-3: 307.7 mW/cm^2^ at 120 °C/30% RH (3× Nafion)
Sulfonated Siliceous Layered Materials (sSLM)	Sulfonic, Layered	Nafion, sPEEK	Water Retention, Proton Conductivity, Thermomechanical Stability	“Bound” Water Structuring, Nacre-like Structure	Nafion N-sSLM5: 30.24 mS cm^−1^ at 120 °C/20% RH
Phosphonated Organosilica Layered Materials (PSLM)	Phosphonic, Layered	sPEEK	Mechanical Strength, Water Retention, Proton Transport	Physical Crosslinking, Grotthuss Enhancement	sPEEK-PSLM3: Conductivity close to Nafion 212 at low RH
Clay-Carbon Nanotubes (Clay-CNT)	Branched 2D/1D Hybrid, Sulfonic	Nafion	Proton Transport, Methanol Barrier	Multi-scale Physical Barrier, Functionalized Network	Nafion/SWy-oxCNT-RSO3H: 7 × 10^−2^ Scm^−1^ at 120 °C/30% RH
sPES/sPEEK Blends	Polymer Blend	sPES	Flexibility, Thermal Resistance, Proton Transport, Methanol Barrier	Miscibility, Complementary Properties	sPES-sPEEK (25/75): 130 mW cm^−2^ at 80 °C/4 M methanol

**Table 2:** Comparative Performance of sPSU-based Membranes for PEMFCs and DMFCs.

Membrane Type	IEC (meq g^−1^)	Water Uptake (wt% at 20–25 °C)	Tensile Strength (MPa)	Young’s Modulus (MPa)	Glass Transition Temperature (Tg, °C)	Proton Conductivity (mS cm^−1^) at 80 °C/90% RH	Activation Energy for Proton Conductivity (kJ mol^−1^)	Water Self-Diffusion Coefficient (D, cm^2^s^−1^) at 130 °C	Methanol Crossover Current (mA cm^−2^) at 80 °C/5 M	Peak Power Density (mW cm^−2^) in Fuel Cell Test (H_2_/O_2_ or DMFC)
Pristine sPSU (Recast)	1.36	27	26.5	15.4	200	69	16.10	1.31 × 10^−7^	379	101 (DMFC)
Pristine sPSU (Extruded)	1.36	22	42.3	34.3	-	78.0 (120 °C)	24.31	1.31 × 10^−7^	-	-
sPSU/LDH	1.49	29	-	-	225	102 (120 °C)	9.25	6.63 × 10^−6^	292	150 (DMFC)
sPSU/sGO	1.32	38	-	-	-	9.4 (80 °C/20% RH)	-	-	-	182.6 (H_2_/O_2_)
sPSU/GO-TiO_2_	1.36	37	-	-	240	100 (100% RH)	-	1.1 × 10^−5^ (130 °C)	-	-

Note: Values for sPSU/sGO and sPSU/GO-TiO_2_ are for DS = 80%. Peak power densities are specified for H_2_/O_2_ or DMFC tests, with relevant T/RH/Concentration.

**Table 3:** Comparative Performance of Selected Advanced Proton Exchange Membranes.

Membrane Type	Host Polymer (If Composite)	Proton Conductivity (mS/cm) (T/RH)	Mechanical Strength (Tensile Strength MPa/Young’s Modulus MPa/Storage Modulus MPa)	Water Uptake (wt%) (T/RH)	Methanol Crossover (mA cm^−2^/Permeability cm^2^s^−1^) (T/Methanol Conc.)	Operating Temperature Range (°C)/High-Temperature Performance Notes	Key Advantages	Key Challenges	Refs.
**PCAM Lab Materials**									
sPSU/LDH	sPSU	4 (90 °C/20% RH); 102 (120 °C/20% RH)	-	29 (20–25 °C); 40 (130 °C)	292 (80 °C/5 M)	Up to 110 °C; 254 mW cm^−2^ at 80 °C/30% RH; 204.5 mW cm^−2^ at 110 °C/25% RH	Cost-effective, high water retention, excellent dimensional stability, high-T operation, reduced methanol crossover	-	[17,19]
Nafion-sGOsulf	Nafion	231.9 (90% RH); 44.9 (90 °C/30% RH)	-	Outstanding retention up to 130 °C	Reduced	High-T operation; internal humidification	Proton superhighways, superior water retention, high conductivity at low RH	-	[21,22]
sPEEK5/SSLM 5 wt%	sPEEK	12.8 (90 °C/30% RH)	68.32 MPa/-/260 MPa	Halved at 5 wt% SSLM	-	Improved water diffusivity at high T	Nacre-like structure, high mechanical strength, high conductivity at low RH, improved hydrolytic stability	Water uptake decline with high filler content	[26,69]
sPES/sPEEK (25/75)	sPES	Superior to pristine sPES at low hydration	Enhanced flexibility, thermal resistance	Water diffusivity 1 order of magnitude higher (50/50 blend)	Dramatically reduced (>3 orders of magnitude vs. pristine sPES)	130 mW cm^−2^ at 80 °C/4 M methanol	Scalable, cost-effective, balanced properties, excellent methanol barrier	-	[34,70]
**Other Advanced Materials**									
Sulfonated Nanocellulose	Cellulose	15 (120 °C, fully hydrated)	1.15 GPa (Young’s Modulus)	6330 (48 h)	8.28 × 10^−9^ (sulfated cellulose)	Up to 190 °C thermal-oxidative stability	Renewable, low-cost, high water uptake, good mechanical robustness, environmentally friendly	Water instability (requires crosslinking)	[11,71]
PA-doped PBIANI	PBI	167 (120 °C/100% RH)	26 ± 3 MPa	-	-	120–200 °C; 691 mW/cm^2^ at 160 °C (PFCB-PBI)	High-T/anhydrous operation, CO tolerance, improved mechanical strength	PA leaching, limited long-term stability	[47]
Sulfonated Graphene Oxide (SGO)	-	1150 (80 °C)	Inferior to Nafion 212 (20.3 MPa tensile stress for Nafion)	Higher than Nafion 212 (0.71 meq g^−1^ IEC for Nafion)	Reduced	-	PFAS-free, high IEC, high proton conductivity, good environmental impact trade-off	Inferior mechanical performance to Nafion	[72]
PIL-PBI Blends	PBI	70 (150 °C)	Lower elastic modulus	-	-	High-T/anhydrous operation	High conductivity at elevated temperatures, non-volatility	IL leaching, limited long-term thermal/mechanical stability	[60]
COF-based PEMs	Various (e.g., polymer composites)	>890 (90 °C/100% RH); 86.3 (160 °C, N2)	Robust structure stability	Pore solvation ability	-	High-T/anhydrous potential	Tunable porosity, ordered channels, high stability, enhanced power density	Humidity dependence, scalability, acid-resistance, MEA integration	[55]
**Commercial Benchmarks**									
Pemion^®^	Sulfo-phenylated Polyphenylene (sPPX-H^+^)	41 (40% RH)	>50/>600		Lower gas	Up to 120 °C; 0.96 W cm^−2^ at 110 °C/50% RH	Exceeded DOE 20,000 cycle AST target (Validated Durability)		[68]
Aquivion SSC-PFSA	-	Higher than Nafion LSC-PFSA (low RH)	Higher crystallinity, higher Tg (140 °C vs. 100 °C for Nafion)	-	-	Up to 110 °C	Higher operating temperature, better performance at low RH, higher stability	-	[44]

**Table 4:** **Analysis of Filler Concentration Regimes and Physical Phenomena**.

Concentration Regime	Physical Phenomenon	Impact on Proton Conductivity	Impact on Mechanical Integrity
**Low Loading (0.1–1.0 wt%)**	**Dispersion Zone:** Nanoparticles are isolated. Formation of interfacial “space-charge” regions.	Slight increase or neutral. Conductivity is dominated by the bulk polymer matrix.	Minimal reinforcement. Fracture toughness may increase due to crack pinning.
**Optimum Loading (1.0–5.0 wt%)**	**Percolation Threshold:** Functional zones around particles overlap, forming continuous conduction pathways.	**Peak Performance.** Rapid increase in conductivity as new high-speed pathways bridge polymer clusters.	Optimal stiffness/toughness balance. Fillers restrict polymer chain mobility (creep resistance).
**Overloading (>5.0–10 wt%)**	**Agglomeration Zone:** Particles clump due to surface energy. Blocking of polymer channels.	**Decline.** Agglomerates act as inert obstacles, increasing tortuosity and severing ionic channels.	Embrittlement. Agglomerates act as stress concentrators, reducing tensile strength and elongation.

**Table 5:** Critical Analysis of Commercialization and Scalability Hurdles for Key PCAM Nanocomposite Strategies.

Nanocomposite System	Primary Commercial Advantage	Key Scalability/Manufacturing Hurdle	Estimated Complexity Cost Barrier (Synthesis)	Long-Term Stability Challenge Addressed
sPSU/Layered Double Hydroxides (LDH)	Cost-effective polymer host, high-T/low-RH operation, effective methanol barrier	Hydrophilic LDH dispersion in polymer matrix; risk of agglomeration; detrimental manufacturing-induced anisotropy (Extrusion Paradox)	Medium (requires controlled particle synthesis and functionalization)	Dimensional instability (via physical crosslinking)
Nafion/Sulfonated Graphene Oxide (sGO)	Proton superhighways, superior water retention, high conductivity at low RH	High cost and complexity of sGO functionalization; difficulty in maintaining homogeneity and preventing agglomeration at industrial scale	High (nanofiller production complexity)	Dehydration/Conductivity collapse at high T
sPEEK/Sulfonated Siliceous Layered Material (sSLM)	Biomimetic ‘nacre-like’ reinforcement, exceptional mechanical durability	Multi-step sol–gel required for designer filler synthesis; difficulty in achieving precise architectural (layered) control during R2R coating	Medium-High (designer filler, complex synthetic route)	Mechanical degradation and swelling (via nacre structure)

## Data Availability

No new data were created or analyzed in this study.
